# Structure-Function Relationships in Pectin Emulsification

**DOI:** 10.1007/s11483-017-9513-4

**Published:** 2018-01-08

**Authors:** F. M. Kpodo, J. K. Agbenorhevi, K. Alba, I. N. Oduro, G. A. Morris, V. Kontogiorgos

**Affiliations:** 1 Department of Food Science and Technology, Kwame Nkrumah University of Science and Technology, Kumasi, Ghana; 2 Department of Nutrition and Dietetics, University of Health and Allied Sciences, Ho, Ghana; 3 Department of Biological Sciences, University of Huddersfield, Huddersfield, UK; 4 Department of Chemical Sciences, University of Huddersfield, Huddersfield, UK

**Keywords:** Pectin, Emulsion, Okra, Interface

## Abstract

The emulsifying characteristics of pectins isolated from six different okra genotypes were investigated and their structure-function relationships have been evaluated. Emulsion formation and stabilization of acidic oil-in-water emulsions (pH 2.0, *φ* = 0.1) were studied by means of droplet size distribution, *ζ*-potential measurements, viscometry, interfacial composition analysis and fluorescence microscopy. Fresh and aged emulsions differed in terms of droplet size distribution, interfacial protein and pectin concentrations (Γ) depending on the molecular properties of pectin that was used. Specifically, pectins with intermediate length of RG-I branching with molar ratio of (Ara + Gal)/Rha between 2 and 3 exhibit the optimum emulsification capacity whereas samples with the molar ratio outside this range do not favour emulsification. Additionally, low amounts of RG-I segments (HG/RG-*I* > 2) improve long term stability of emulsions as opposed to the samples that contain high amounts of RG-I (HG/RG-*I* < 2) which lead to long term instability. Protein was not found to be the controlling factor for the stability of the dispersions. The present results show that rational design of pectin should be sought before application as functional ingredient in food and/or pharmaceutical systems.

## Introduction

Industrial demand for natural ingredients has increased in recent times and propelled the need to explore properties of pectins from different sources for new technological functionality in emulsions [1, 2]. Many food products require emulsifiers to stabilize the oil-water droplets dispersed in a continuous aqueous phase. Emulsifiers are surface-active agents that facilitate emulsion formation by reducing the interfacial tension between the two immiscible phases. Proteins are commonly used as food emulsifiers because they possess suitable molecular characteristics (*e.g*., molecular weight, conformation, flexibility, and polarity) so as to unfold and adsorb at the oil-water interface. As a result, protein-stabilized emulsions show negligible changes in droplet size distribution over time [3]. Most polysaccharides, however, because of their reduced hydrophobicity and high molecular weight are usually not utilized as emulsifying agents or require some form of modification before application [4]. However, the emulsifying capacity of pectins extracted from sugar beet [5–7], pumpkin [8], citrus [9, 10], hawthorn [1], and okra have long been demonstrated [11, 12].

Pectin is a heteropolysaccharide of particular industrial importance with diverse functionality due to its tunable structure. It consists of two major blocks, namely, homogalacturonan (HG) and rhamnogalacturonan I (RG-I). Homogalacturonan is the most abundant polymeric segment (~ 65%) and is composed of long chains of linearly 1 → 4-linked α-D-GalpA residues (galacturonic acid) [13]. Some of the carboxyl groups are methyl-esterified at C-6 position and pectins with degree of methyl esterification greater than 50% are known as high methyl-esterified (HM) and those with lower than 50% as low methyl-esterified (LM). It is possible that pectin is also acetyl esterified at O-2 and/or O-3 positions of GalpA. The HG segment is commonly referred to as the “smooth” region of pectin because of the absence of branching. The rhamnogalacturonan I (RG-I) is composed of the repeating disaccharide galacturonic acid and rhamnose [α-(1 → 2)-D-GalpA–α-(1 → 4)-L-Rhap]_n_ where n can be greater than 100. Rhamnose units on RG-I backbone contain polymeric side-chains predominantly composed of the neutral sugars arabinose, galactose or both. As a result, this region is also known as the “hairy” part of pectin [14]. Other moieties may be observed depending on the botanical origin and method of extraction such as rhamnogalacturonan II (RG-II), arabinogalactan, arabinan, apiogalacturonan or xylogalacturonan. Proteins may also be attached to side chains of RG-I regions further contributing to the complexity of the structure.

The emulsifying properties of sugar beet and okra pectins have generally been attributed to either high acetyl content, presence of ferulic acids, or covalently bound proteins [7, 11]. In addition, side chains have been also shown to play role on the emulsification capacity and stability suggesting that RG-I containing pectins could have improved emulsifying properties as opposed to pectins with linear backbone [15, 16]. Consequently, pectins from citrus and apple have not been considered as useful emulsifiers due to the low protein and acetyl group contents and lack of extended branching [7]. In addition to the high levels of acetyl groups and presence of protein moieties, some pectins also have the ability to increase the viscosity of the continuous aqueous phase and reduce the movement of oil droplets, which suppresses phase separation and increases emulsion stability [2].

In our previous investigations, it was shown that okra pectin can successfully stabilise acidic oil-in-water emulsions [11, 12]. Furthermore, pectins isolated from six different okra genotypes have been characterized showing diversity in structure, and composition [17]. As a result, we were lead to form a hypothesis that okra presents a source of pectin that can be tailored to achieve adjustable functionality. Therefore, the objective of the present study, is to investigate the emulsifying properties of pectins with different molecular architectures and arrive at structure-function relationships that can be used to design pectins with optimal interfacial properties.

## Material and Methods

### Materials

Pectin was isolated from six okra genotypes (Asha, Agbagoma, Asontem, Balabi, Sengavi and Penkrumah) were cultivated in Ghana and extracted using the protocol, as reported previously [17, 18]. All chemicals used were of analytical grade and were purchased from Sigma-Aldrich (Poole, UK). De-ionized water was used throughout the experiments.

### Preparation of Emulsions

The capacity of the six okra pectins to act as emulsifiers was tested by means of emulsifying sunflower oil into an aqueous buffer at pH 2.0 (100 mM KCl/HCl) containing 1.67% *w*/*v* of pectin so as to yield emulsions of *φ* = 0.1 and of a nominal pectin concentration in the entire emulsion volume of 1.5% w/v. Emulsions were fabricated at room temperature in two stages first by obtaining pre-emulsions with a high-speed homogenizer for 2 min (IKA T18 basic, Ultra-Turrax, Staufen, Germany) followed by further emulsification using a Model UP 100H ultrasound device (Hielscher Ultrasonics, Teltow, Germany) with a MS7 tip at 30 kHz for 40 s with pulsed ultrasound (30% per second) at 100% amplitude.

### Determination of Droplet Size Distribution

In order to quantify the capacity of these emulsifiers with regard to long-term emulsion stability, the droplet size distribution and the average droplet sizes were measured at set time intervals (0, 5 and 15 days) using a Malvern Mastersizer 2000 (Malvern Instruments Ltd., Malvern, UK) laser diffraction particle size analyzer using the small volume sample dispersion unit Hydro 2000SM (Malvern Ltd., UK). Refractive index of sunflower oil and the dispersion medium (HCl/KCl buffer, pH 2) were set to 1.435 and 1.333, respectively. Consequently, droplet sizes of the emulsions were described using the surface-weighted mean diameter (d_32_) and volume-weighted mean diameter (d_43_) [12].

### Interfacial Composition Analysis

The interfacial composition of formulated emulsions was characterized in terms of adsorbed protein and pectin at the oil-water interface. First, okra pectin stabilized emulsions were centrifuged at 3000 x g for 5 min (Centrifuge 5702, Eppendorf, Hamburg, Germany) in order to separate the dispersed phase (oil droplets) from the continuous phase (serum). The serums were then carefully collected using a syringe. Concentrations of protein and pectin (expressed as total carbohydrate) were measured in pectin solutions (*i.e*., aqueous phase before emulsification) and in serums according to Bradford method [19] or phenol-sulphuric acid assay [20, 21]. Interfacial protein and pectin concentrations (Γ, mg m^−2^) were calculated as protein or pectin concentration difference between the pectin solution and serum divided by the specific surface area (SSA, m^2^ mL^−1^) of the oil droplets that was obtained from the instrument:

1 $$ \Gamma =\frac{mg\  of adsorbed protein or pectin}{SSA\times mL\  of oil in emulsion} $$

All ζ-potential measurements were performed using a ZetaSizer Nano Series ZEN2600 (Malvern Instruments, Malvern, UK) at 25 °C. Emulsions were diluted 1000 times in buffer in order to avoid multiple scattering effects. Refractive indices were set as described in the previous section. All measurements were performed in triplicates immediately after emulsion preparation and after 0, 5 and 15 days of storage.

### Rheological Measurements

Rheological properties of emulsions were measured using a Bohlin Gemini 200HR nano rotational rheometer (Malvern Instruments, Malvern, UK) equipped with cone-plate geometry (40 mm diameter, cone angle 4°). Viscosity measurements were performed on the fresh emulsions, and during storage (after 5 and 15 days). All measurements were completed in a steady shear mode in the range of 0.1–1000 s^−1^ at 25 °C.

### Emulsion Morphology

Fluorescent microscopy was performed using Imager Z1 AxioCam MRm camera supported by ZEN 2011 software (Carl Zeiss Microscopy GmbH, Göttingen, Germany). The fluorescent dye (Rhodamine B, 0.02%) was placed in the pectin solutions prior to emulsification. Emulsions were placed on a glass slide and covered with a coverslip prior to imaging.

## Results and Discussion

### Emulsification Capacity of Pectin and Long Term Stability

Pectin samples used in the formulation of emulsions have been previously characterised and key molecular characteristics that are relevant to the present work are reproduced in Table 1 [17]. In brief, all samples had low degree of methylation (*i.e.*, LM-pectin), high degree of acetylation (DA), and were of high molecular weight. All samples had high total carbohydrate content and comparable amounts of protein. This is an important observation, as any differences in the emulsification capacity can be attributed to the variations in the molecular structure of pectin rather than to protein content. The molar ratio of (Arabinose + Galactose)/Rhamnose (R, Table 1) that was calculated from the monosaccharide composition of pectins is an indication of the size of the branching of the side chains in the rhamnogalacturonan-I (RG-I) units with higher values indicating greater branching. Samples can be grouped into pectins with short (*R* < 2, Asha, Penkruma), intermediate (2 < *R* < 3, Sengavi, Balabi), or long branches (*R* > 3, Asontem, Agbagoma). It should be also mentioned that pectins presented a diversity in the amount of RG-I component. For instance, Asha and Balabi are rich in RG-I domains making them “hairy” pectins whereas Penkruma, Asontem, Agbagoma and Sengavi are essentially “smooth” pectins (HG/RG-I ratio in Table 1).

**Table 1:** Molecular characteristics of pectin samples that have been used as emulsifiers

Genotype	Protein (% d.b)	Total Carbohydrate (% d.b)	D-GalA (% d.b)	DM (%)	DA (%)	(Ara+Gal)/Rha (R)	RG-I	HG/RG-I	[*η*] (dLg^−1^)	M_w_ (× 10^3^ gmol^−1^)
Asha	5.5 ± 3.1^a^	86.3 ± 2.0^a^	63.4 ± 1.1^a^	17.2 ± 1.4^a^	39.3 ± 4.3^a^	1.9	42.7	1.3	4.35	1202
Penkruma	4.4 ± 1.4^a^	87.4 ± 1.0^a^	62.4 ± 4.7^a^	17.0 ± 0.1^a^	19.9 ± 0.5^c^	1.6	23.6	3.1	4.35	893
Asontem	3.8 ± 1.8^a^	72.5 ± 2.5^b^	54.2 ± 4.6^b^	20.4 ± 1.8^bc^	40.1 ± 5.7^a^	3.4	29.8	2.2	3.55	1233
Agbagoma	5.4 ± 2.8^a^	66.2 ± 1.0^b^	51.9 ± 3.4^b^	20.9 ± 1.8^c^	31.7 ± 5.5^b^	3.9	27.2	2.5	3.56	1419
Sengavi	7.1 ± 2.4^a^	66.2 ± 4.3^b^	59.2 ± 1.0^ab^	18.4 ± 1.4^ab^	22.4 ± 3.3^c^	2.8	28.5	2.3	5.10	1693
Balabi	3.3 ± 1.0^a^	87.5 ± 3.5^a^	42.8 ± 1.3^c^	25.5 ± 1.8^d^	37.9 ± 5.8^ab^	2.4	41.1	1.3	2.91	791

*D-GalA* galacturonic acid, *DM* degree of methylation, *DA* degree of acetylation. Sugar molar ratio (Ara + Gal)/Rha is an indication of the size of the branching of side chains of RG-I with higher values indicating greater branching. [*η*] is the intrinsic viscosity and M_w_ the weight average molecular weight of the samples. All the data are reproduced with permission from Kpodo et al. [17]

*d.b* Dry weight basis. Means sharing the same letters in a column are not-significantly different (*p* > 0.05)

**Fig. 1:**
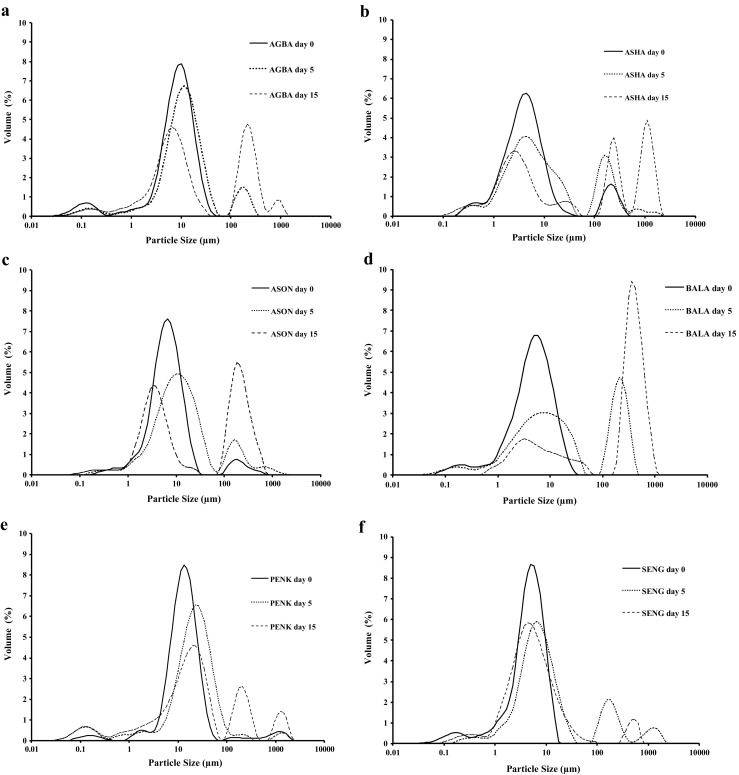
Particle size distribution of emulsion prepared using different okra pectins: **a** Agbagoma, **b** Asha, **c** Asontem, **d** Balabi, **e** Penkruma, and **f** Sengavi

The ability of the isolates from different genotypes to stabilize emulsions was determined by monitoring the average droplet sizes at set time intervals for a period of fifteen days. Differences were observed for the average droplet diameter of the emulsions (Table 2; Fig. 1). The surface weighted mean diameter (d_3,2_) for the fresh emulsions ranged from 1.3 to 3.7 μm thus demonstrating good emulsifying capacity for all samples studied. Fresh emulsions prepared with pectins from the genotypes Sengavi (1.4 μm) or Agbagoma (1.3 μm) recorded the lowest initial d_3,2_ value whereas those stabilized with Penkruma (3.5 μm) or Asontem (3.7 μm) were at the higher end of the range of sizes. Emulsion destabilization was evident after the fifth day in all samples and continued throughout the storage period as demonstrated by the progress of the volume weighted mean diameter (d_4,3_). Instability in bimodal dispersions is usually controlled by the higher modes resulting in the predominance of coalescence as the major destabilisation mechanism [22]. All samples developed a second mode during ageing, however, Sengavi pectin-stabilized emulsions showed minimal destabilization compared to those prepared with pectin from the other okra genotypes (Fig. 1). Volume weighted mean diameter (d_4,3_) for emulsion samples containing Sengavi pectins remained relatively stable after five days of storage (around 30 μm) without particular development of the higher modes. Hence the Sengavi-stabilized emulsions exhibited the highest stability during storage compared to emulsions prepared with the other pectin types. On the contrary Asha-stabilized emulsions showed poor performance in terms of preventing droplet growth during ageing.

**Table 2:** Effect of okra pectin genotype and storage time on average droplet diameters and ζ-potential of oil-in-water emulsions

Sample	Time (days)	d_32_ (μm)	d_43_ (μm)	Span	ζ-Potential (mV)
Asha	0	2.2 ± 0.0	25.3 ± 0.7	28.9 ± 0.2	−2.7 ± 0.1
	5	2.6 ± 0.4	60.9 ± 9.6	32.8 ± 8.1	−3.2 ± 0.2
	15	1.8 ± 0.8	161.3 ± 0.3	75.8 ± 7.7	−1.8 ± 0.5
Penkruma	0	3.5 ± 0.0	51.7 ± 0.0	1.9 ± 0.0	−4.6 ± 0.2
	5	1.3 ± 0.0	51.8 ± 0.0	2.7 ± 0.0	−5.5 ± 0.7
	15	1.2 ± 0.1	105.0 ± 4.8	13.1 ± 1.7	−2.7 ± 0.5
Asontem	0	3.7 ± 0.0	20.5 ± 0.0	2.4 ± 0.0	−3.0 ± 0.0
	5	4.6 ± 0.3	53.9 ± 0.5	14.5 ± 1.9	−3.2 ± 0.1
	15	2.8 ± 0.1	112.1 ± 4.6	18.9 ± 8.1	−1.0 ± 0.2
Agbagoma	0	1.3 ± 0.1	10.8 ± 0.4	2.0 ± 0.0	−3.2 ± 0.0
	5	1.8 ± 0.1	23.5 ± 3.3	3.0 ± 0.6	−3.3 ± 0.3
	15	1.6 ± 0.3	103.8 ± 1.7	31.3 ± 3.1	−2.0 ± 0.0
Sengavi	0	1.4 ± 0.0	4.8 ± 0.0	1.8 ± 0.0	−2.4 ± 0.0
	5	3.6 ± 0.4	38.6 ± 0.0	22.5 ± 3.9	−2.7 ± 0.1
	15	2.2 ± 0.0	32.8 ± 0.0	4.5 ± 0.0	−1.8 ± 0.4
Balabi	0	2.0 ± 0.5	5.7 ± 0.2	2.3 ± 0.1	−3.0 ± 0.7
	5	1.8 ± 0.0	72.5 ± 0.1	18.4 ± 2.4	−3.1 ± 0.7
	15	1.8 ± 0.2	115.8 ± 0.2	8.3 ± 1.4	−1.7 ± 0.1

**Fig. 2:**
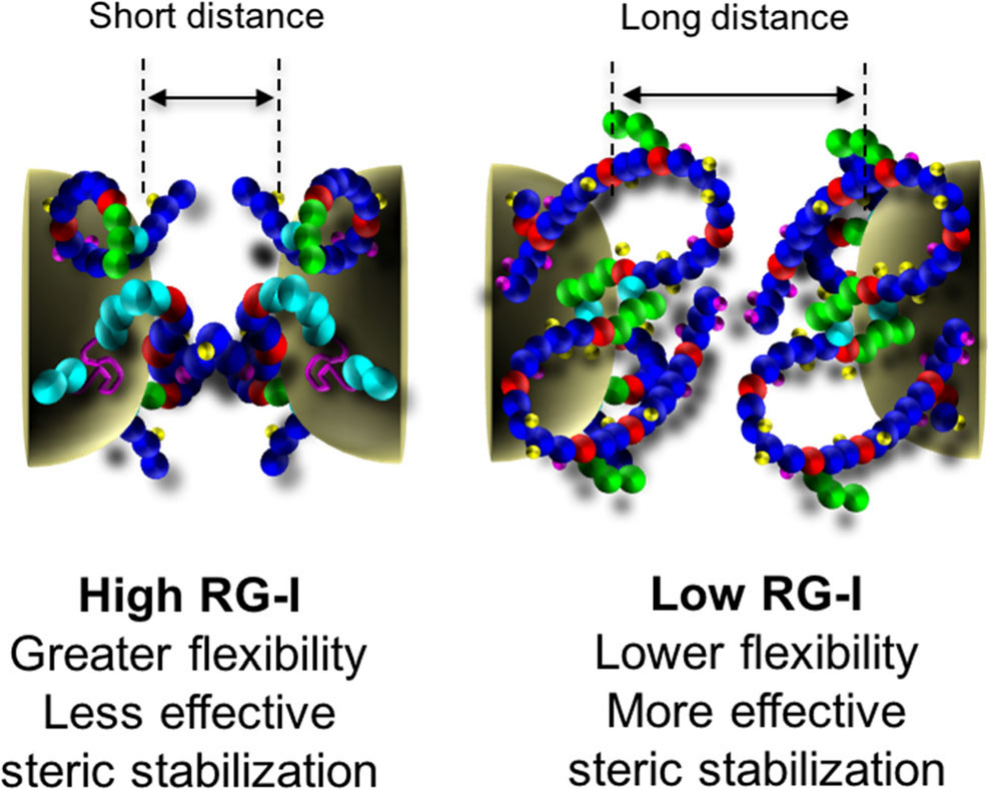
Schematic depiction of the influence of RG-I segments on stability of pectin-stabilised emulsions. Pectins with high amount of RG-I have greater chain flexibility resulting in shorter distances between droplets compared to those with low RG-I content leading to less effective steric stabilisation

A close inspection of the behaviour of the temporal evolution of droplet growth reveals two characteristic tendencies that can be linked to the fine structure of pectin. The first trend is related to that of the fresh emulsions (*i.e.*, day zero) and droplet size follows the order of increasing d_4,3_ which was Sengavi < Balabi < Agbagoma < Asontem < Asha < Penkruma. The second trend refers to the order of droplet sizes after fifteen days of storage, importantly from an application point-of-view this was different than that of fresh emulsions (Sengavi < Agbagoma < Penkruma < Asontem < Balabi < Asha). This observation confirms that the initial droplet size and span of the size distribution curves together with the fine structure and molecular characteristics of pectin control the long-term stability of the emulsions. Careful examination of the molecular properties of the samples disclose the importance of branching (*i.e*., R ratio, Table 1) and RG-I content (Table 1). Pectin samples with intermediate length of branching (*i.e.*, 2 < *R* < 3, Sengavi, Balabi) contribute favourably to the emulsifying capacity, which refers to the droplet size of fresh emulsions (*i.e.*, small d_4,3_ and d_3,2_ at day zero). Conversely, short (*R* < 2, Asha, Penkruma) or long branching (*R* > 3, Asontem, Agbagoma) do not assist emulsification. These results are in agreement with previous observations of okra pectin stabilised emulsions where pectin with intermediate R values resulted in favourable emulsification of hydrocarbons [12]. The second observation relates to the stability of emulsions after fifteen days of storage. It appears that samples with lower RG-I content (Penkruma, Agbagoma, Sengavi, Asontem) exhibit greater long-term stability in comparison to those with higher (Balabi, Asha). It has been shown that RG-I segments impart greater flexibility to the pectin chains [23]. In particular, persistence length that can used as a measure of chain flexibility has been found to be lower for okra pectin chains with greater content in RG-I regions [24]. Consequently, the additional flexibility of the chains creates shorter separation distances between droplets resulting in ineffective steric stabilization compared to those with lower amounts of RG-I (Fig. 2). In addition, higher flexibility may make it easier for pectin to desorb from the interface as okra pectin does not form highly interconnected and elastic networks at the interface, as is often observed in pure protein stabilized interfaces [12], thus resulting in lower overall stability of emulsions during ageing. It should be also mentioned that no clear relationship was observed between molecular weight and long-term stability. For instance, sample Pekruma with low molecular size presents excellent long-term stability compared to Asha with a considerably higher molecular weight. Nevertheless, pectins containing RG-I units result in better overall emulsification performance than those with linear backbone (*e.g.*, Penkruma *vs.* Asha), a congruent observation with previously published work [5, 15]. A link between the length of branching of RG-I units has not been previously established and the present work reveals that pectins with intermediate branching (2 < *R* < 3) and lower RG-I content result in optimum emulsification thus providing insights into the mechanisms by which pectins stabilise oil-in-water emulsions. The ability of pectin to sterically stabilize oil droplets is attributed to the branching of RG-I domains, whereas electrostatic stabilisation originates from HG-domains due to the ionization of carboxylic groups [16]. In the present system, the carboxyl groups of the D-GalA are not ionised as the pH of the emulsions is low (pH 2.0) also seen in the low *ζ*-potential values (Table 2), and as a result electrostatic stabilisation is not the dominant mechanism of stabilisation in the present systems. In addition, steric stabilisation of this particular type of pectin could be enhanced by the presence of hexameric self-assembled structures, consisting of smaller macromolecular entities held together by hydrogen bonding [25], that may form at the interface. The presence of these neutral sugar side-chains and proteinaceous moieties contribute to the long-term emulsion stability due to the formation of interfacial layers thus providing effective steric stabilisation that impedes emulsion coarsening [26, 27]. In the present systems, protein contents were not significantly (*p* > 0.05) different between samples and although it contributes to formation and stability of the emulsions it is not the determinant factor. In order to address this, examination of the interfacial composition provides further evidence on the role of protein and pectin in the differences in emulsion stability is explored in the next section.

### Interfacial Composition Analysis

The interfacial composition of emulsions stabilized with pectins from different okra genotypes was assessed in order to better understand mechanisms of interfacial adsorption and emulsifying action of studied pectins (Table 3). The results show that although a larger proportion of proteins (~80%) was adsorbed at the interface than pectins (~50%), the interfacial concentration of pectins was higher (0.6–3.6 mg m^−2^) than that of the proteins (0.3–1.0 mg m^−2^) for all emulsions studied, in line with our previous observations [12]. It should be mentioned that it is unknown whether protein is integral part of pectin or a mere contaminant during the extraction process, as protein measurements cannot distinguish between the two cases. Consequently, adsorbed pectin fractions are treated as either being rich in protein or being adsorbed *via* a bi-layer adsorption mechanism, as it is discussed below. The surface coverage of pectins in this study was lower than the values previously reported for okra pectin (9.4 mg m^−2^) [12], sugar beet pectin (7.5 mg m^−2^) [28], or citrus pectin (9.8 mg m^−2^) [ 29]. However, among the samples studied, pectin from Asontem demonstrated the highest surface coverage in terms of pectins (3.6 mg m^−2^) and protein (1.0 mg m^−2^) whereas Penkruma (0.9 mg m^−2^) and Agbagoma (0.6 mg m^−2^) showed the lowest pectin surface coverage. It should be noted that no particular trend can be observed between interfacial load and stability. For instance, Sengavi which is the most stable emulsion has lower Γ_pectin_ and Γ_protein_ than Asontem showing that pectin structure plays important role for stability. Nevertheless, the extent of protein and pectin adsorption at the interface generally supports the bilayer steric stabilization mechanism with pectin being the predominant biopolymer at the interface*.* Typically, the amount of adsorbed biopolymer at the interface which is consistent with monolayer coverage is in the order of 1 mg/m^2^. Consequently, Asontem forms bilayers with both protein and pectins, Asha, Sengavi, and Balabi form monolayers only with pectin whereas Penkruma and Agbagoma do not seem to form monolayers with any of the biopolymers. In systems where protein and polysaccharides are present at the same time, proteins attach through multiple anchor points to the surface and form a relatively thin interfacial layer. Pectin attach either directly at the interface or at the pre-formed protein interfacial layer (bi-layer) thus creating an effective steric barrier that prevents coalescence. Additionally, the presence of hydrophobic groups such as acetyl and methyl may contribute to pectin ability to adsorb at the lipid surfaces while the hydrophilic chains of pectin extend into the aqueous phase providing stability against droplet aggregation through steric hindrance, as described earlier [16]. *ζ*-Potential measurements also show some evidence that the interface may be predominantly occupied by pectin, as the interfacial charge is negative for all emulsions (Table 2) and remained constant after fifteen days of storage albeit at the limits of instrumental resolution. It should be mentioned that at low pH (2.0), proteins mainly carry positive charge and if they were the sole residents of the interface the charge would be positive.

**Table 3:** Percentage of adsorbed pectin and protein, and interfacial pectin and protein concentration (Γ) of fresh emulsions stabilized with okra pectin from different genotypes

Sample	Adsorbed pectin (%)	Adsorbed protein (%)	Γ_pectin_ (mg m^−2^)	Γ_protein_ (mg m^−2^)
Asha	47.7 ± 1.3^b^	78.2 ± 0.1^ab^	1.9 ± 0.1^c^	0.7 ± 0.0^b^
Penkruma	55.4 ± 0.5^a^	82.2 ± 5.3^ab^	0.9 ± 0.0^e^	0.3 ± 0.0^c^
Asontem	49.0 ± 1.5^b^	89.9 ± 8.2^a^	3.6 ± 0.1^a^	1.0 ± 0.1^a^
Agbagoma	41.5 ± 1.3^c^	74.8 ± 6.4^b^	0.6 ± 0.0^f^	0.4 ± 0.0^c^
Sengavi	59.5 ± 2.5^a^	46.0 ± 3.2^c^	1.5 ± 0.1^d^	0.3 ± 0.1^c^
Balabi	50.1 ± 3.3^b^	56.2 ± 7.1^c^	2.3 ± 0.2^b^	0.3 ± 0.0^c^

Values indicate the mean ± SD of triplicates. Mean values with different letters in a column are significantly different (*p* < 0.05)

### Emulsion Rheology and Microstructure

**Fig. 3:**
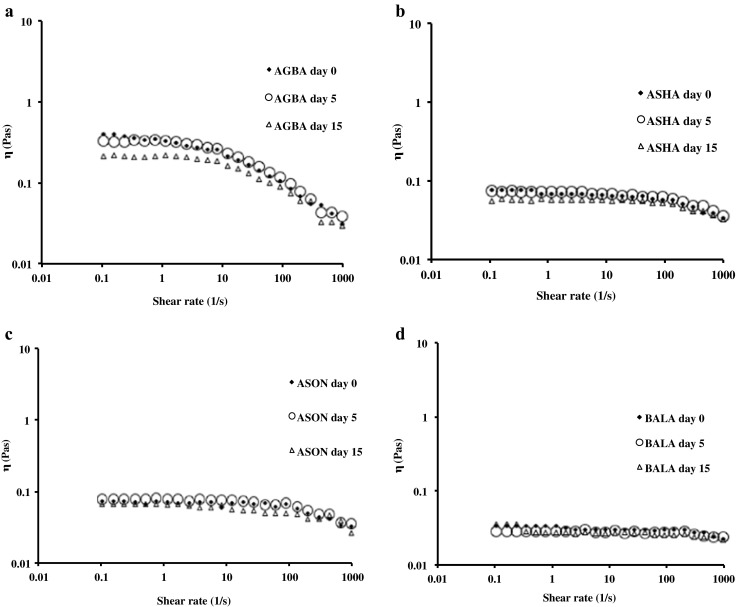
Typical rheological characteristics of different okra pectin emulsions: **a** Agbagoma, **b** Asha, **c** Asontem, and **d** Balabi

**Fig. 4:**
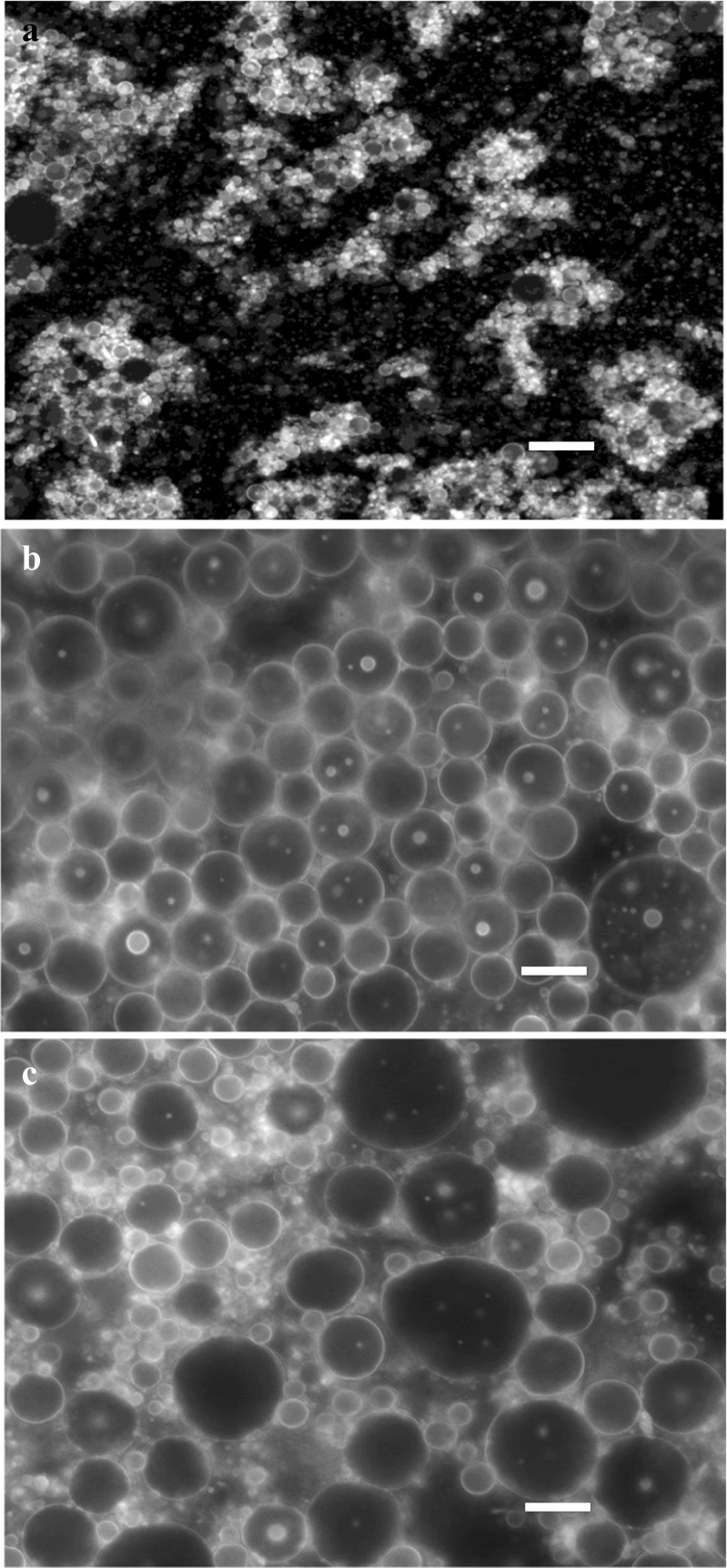
Typical fluorescent microscopic image of undiluted okra pectin stabilized emulsions at **a** day 0, **b** day 5 and **c** day 15. Scale bars are 100 μm

The final step in the present investigation was to examine the rheological and microstructural properties of emulsions that could give insights on the level of interactions between the droplets. Viscosity curves of the emulsions exhibited flows ranging from Newtonian to shear thinning that followed the molecular weight of the samples (Table 1). For instance, Balabi stabilized emulsions showed Newtonian behaviour whereas emulsions stabilized with other pectins demonstrated shear thinning behavior that progressively increased with molecular weight of pectins (*e.g*., *η*_Asontem_ < *η*_Agbagoma_) (Fig. 3). A critical observation of the shear rate sweeps for all the emulsions is that their viscosity does not change with ageing. A variety of factors can influence the rheology of emulsions during storage and this includes the nature of the continuous and dispersed phase, droplet-droplet interactions, and the droplet size [30]. Constant viscosity throughout the storage period indicates limited weak flocculation. Additionally, increase in viscosity during storage can be caused by pectin desorption from the interface thus increasing the viscosity of the continuous phase. Results indicate that any possible desorption of pectin during 15 days of storage did not have any measurable changes in emulsion viscosity, which is in contrast to our previous report [11] perhaps due to structural differences of the samples. However, microscopic morphology of the emulsions obtained under quiescent conditions reveal clusters of flocculated droplets (Fig. 4). The bright areas around the droplets represent adsorbed protein that becomes less intense with ageing due to redistribution of protein in larger surface area, as the droplets increase in size. It is evident that although some flocculation exists in the undisturbed samples, the intermolecular forces are not strong and the droplets disperse under the influence of the shear field particularly for the emulsions stabilized with the low molecular weight samples.

## Conclusions

The role of pectin macromolecular structure on its emulsifying capacity has been investigated. It has been shown that macromolecular characteristics of pectin influence the emulsification capacity and long-term stability performance of emulsions at acidic environments. Specifically, the prevalence of RG-I segments and the length of their branches have been shown to influence emulsion stability. In particular, branches of intermediate length with molar ratio of (Ara + Gal)/Rha ranging between 2 and 3 exhibit the optimum emulsification capacity. On the contrary, short ((Ara + Gal)/Rha < 2) and long branches ((Ara + Gal)/Rha > 3) do not favour emulsification. In addition, low amounts of RG-I (HG/RG-*I* > 2) segments improve long term stability of emulsions as opposed to the samples that contain high amounts of RG-I (HG/RG-*I* < 2) leading to long-term instability. Protein, which is inevitably present, although it may contribute to emulsion formation, it is not the predominant factor responsible for emulsion stability and overall physicochemical behaviour. The present work demonstrates to our knowledge for the first time the link between fundamental structural properties of pectin with its interfacial functionality at low pH environments. Results show that to arrive to the desired functionality the first step should be the selection of the pectin with appropriate molecular characteristics.
